# Microbial enzymes in the Mediterranean Sea: relationship with climate changes

**DOI:** 10.3934/microbiol.2019.3.251

**Published:** 2019-09-12

**Authors:** Renata Zaccone, Gabriella Caruso

**Affiliations:** Institute of Polar Sciences, ISP–CNR, Spianata S. Raineri 86-98122 Messina, Italy

**Keywords:** enzymatic activities, biogeochemical cycles, climate change, Mediterranean Sea

## Abstract

In most of the aquatic ecosystems, microorganisms are major players in the biogeochemical and nutrients cycles (Carbon Nitrogen, Phosphorus), through their enzymatic activities (leucine aminopeptidase, alkaline phosphatase and beta-glucosidase) on organic polymers such as polypeptides, organophosphate esters and polysaccharides, respectively. The small monomers released by decomposition are metabolised by microbes, supporting their growth. Most of the extracellular enzymes are adaptative and their synthesis and activity is strongly affected by environmental factors, consequently the relative importance of leucine aminopeptidase, alkaline phosphatase and beta-glucosidase reflects differences in the composition of organic matter and assume a different meaning.

Since more than two decades, at the CNR the influence of climate changes, seasonal variability, depth and coastal input on the patterns of enzymatic activities in the Mediterranean Sea have been studied. Its particular characteristics of a semi-closed basin, high summer evaporation and the occurrence of important water dynamics, make this ecosystem particularly suitable as a model site for climate changes-related observations.

The present paper reviews the current information of environmental changes on extracellular enzymatic activity obtained in the Mediterranean areas with the aim of evaluating the effects of environmental changes on the microbial activities. The obtained results revealed significant variations in the rates of hydrolytic activities in relation to space and time, with the highest levels generally found in the epipelagic layer (0–100m) and in coastal zones during warm periods. In the Central Mediterranean Sea their relationship with temperature changes was demonstrated.

Spatial variations in the relative enzyme activities also suggested a modulation in the metabolic profiles of the prokaryotic communities, with biogeochemical implications in nutrient regeneration.

Long term studies on microbial activity and abundances in relation with rising temperatures can have a predictive value to describe the evolutionary scenario of microbial processes and the response of microbial metabolism to climate changes in the Mediterranean Sea.

## 1. Introduction

Marine microorganisms are the most abundant organisms in the oceans; in spite of their small size, they account for a significant fraction of living planktonic biomass and play a major role in biogeochemical processes in aquatic ecosystems [1]. In oligotrophic water bodies the role of microorganisms becomes even more important, because in such environments with low nutrient concentrations, prokaryotic heterotrophs compete with phytoplankton for the uptake of the low amounts of available nutrients, mostly derived by regeneration processes [2].

Heterotrophic bacteria contribute to nutrient cycles through the production of new bacterial biomass (bacterial carbon production) which acts as a temporary Carbon storage, influencing the amount of particulate organic matter (POM) available to higher trophic levels or exported to deeper waters. In fact, through the carbon uptake, prokaryotes convert the organic matter from dissolved to particulate phase, creating ‘food’ for large sized organisms within the ‘Microbial loop’ [3].

Large biomasses of photosynthetic microorganisms present in the oceans remove huge quantities of atmospheric carbon. Then, through the Microbial Carbon Pump mechanism [4],[5], microorganisms export carbon to the deep ocean, producing long-lived organic carbon. The ability of microorganisms to produce or degrade this carbon is expected to change according to the variations of environment in which the microbes are living.

Understanding how complex organic matter is transformed and remineralized by marine heterotrophs, and how this activity varies geospatially, is thus fundamental to our knowledge of the microbially-mediated carbon cycle on a global scale.

The organic matter present in marine waters has a heterogeneous matrix, composed of some labile materials such as proteins, simple sugars and fatty acids and other more refractory compounds, less prone to degradation. Indeed, heterotrophic bacteria can assume directly molecules with low molecular weight (below 600 daltons such as amino acids, simple sugars, fatty acids), sufficiently small to be transported across cell membranes, while organic polymers require to be first hydrolyzed into monomers or low- molecular weight compounds before their incorporation into the bacterial cell [6],[7]. The enzymatic hydrolysis of organic polymers by means of specific enzymes is generally considered as a key step in regulating organic matter utilization and its further processing [8].

Describing the variability in extracellular enzymatic activity in the marine environment, and studying the factors that drive this activity, is necessary to understand the global dynamics of the carbon cycle [2],[8],[9].

The Mediterranean Sea is generally recognized to be an oligotrophic basin with several distinctive features such as high summer evaporation and limited freshwater input, which make it particularly sensitive to climate changes [10]. Its main characteristics are:

- a decreasing nutrient concentration from West to East, with increasing oligotrophic conditions in the Eastern basin [10]. The levels of phosphate are in fact the main limiting factor for biological productivity in the eastern Mediterranean,
- the presence of Atlantic waters with low salinity and temperature in the West basin and the intense water evaporation in the Levantine basin [11],[12],
- a relatively warmer (about 13 °C) water in the deep zones compared to deep oceans,
- the prevalence of small heterotrophs and small phototrophs with a microbially dominated food web [13],[14],
- the presence of Levantine Intermediate Waters [at about 300–400 m depth] and dense water formation phenomena that occur in specific sites such as the Gulf of Lions, the Adriatic Sea, and the Aegean Sea [15],[16].

## 2. The climate change

Marine ecosystems play an important role in regulating climate, since they are a reservoir of 50% of anthropogenic carbon emissions, but at the same time, climate change affects natural systems. Understanding how microbial communities are affected by multiple climate change drivers is important to make accurate predictions of ecosystem evolution in response to changing climate scenarios [17].

Studies are currently performed to show that marine microbes could serve as sentinels to monitor climate change effects on marine ecosystems. Key ecosystem processes involved in Carbon cycle, like bacterial production, respiration, bacterial growth and losses by viral lysis and grazing, trophic interactions, all regulated by temperature, are likely to change in a warmer ocean, but the direction and the amplitude of phenomena are still a matter of debate [18]. This is particularly true for the Mediterranean Sea, reported to be one of the most oligotrophic ecosystems in the world [10].

Global warming is likely to reach 1.5 °C between 2030 and 2052 if it continues to increase at the current rate [19]. Many land and ocean ecosystems and some of the services they provide have already changed. The Mediterranean Sea levels will change and its circulation patterns will be modified [20].

Recent studies have reported a warming trend throughout the Mediterranean Sea, with temperature increases ranging from 0.017 °C yr^−1^ (in the mid-western Ionian sub-basin) to 0.05 °C yr^−1^ (north-east of the Levantine sub-basin). In the western Mediterranean Sea an increase of 0.032 °C yr^−1^ was reported; seasonal changes and spatial differences among its sub-basins were also observed [21].

The climate change occurring in the Mediterranean Sea may produce a number of evident direct and indirect effects on this marine ecosystem. The rapid heating of the water, especially since the '80s, is profoundly changing plankton biodiversity and food webs due to variations in the abundance and distribution of species [13]. Furthermore, as a direct effect, global warming may result in increased surface stratification, causing changes in thermohaline circulation and stimulating the rates of biological processes [18],[22]. However, predicting the effects on microbial abundance and metabolism of a warmer ocean is a current hot topic issue and the magnitude and implications of microbial response to climate changes remain still mostly unknown [17],[23],[24].

Sea temperature increase could influence the metabolism of heterotrophic microorganisms, causing cause changes in species distribution and in patterns of extracellular enzymatic activities that act both in surface and in deeper ocean layers.

Indeed, in the Mediterranean basins, increased enzymatic activities and decomposition rates have recently been observed [2012–2013] in the surface layer in coincidence with summer water warming [25],[26]. The Authors hypothesized that an important link between temperature and microbial enzymatic activities occurred in the surface layer of this area, with high amounts of organic matter utilized by prokaryotes mostly in warm periods, similarly to what found in other temperate regions by Hoppe et al. [27].

Other environmental factors that co-vary with temperature, such as nutrient concentration or primary productivity may themselves play a larger effect on bacterial activity [17],[18]. In fact, different environmental variables can influence each one of the microbial size components of food web and consequently the processes associated with global changes could be accelerated or compensated.

To date, no report on Mediterranean microbial community response to ocean acidification is available; in mesocosm experiments the effect of an increase in dissolved CO_2_ on the microbial communities in two opposite seasons was studied [28],[29].

Ocean acidification caused by the rising of CO_2_ produces changes in water pH; enzymes are sensitive to pH changes as they exhibit their activity in a specific pH range. However, the extracellular enzymes leucine aminopeptidase and glucosidase were not found to show significant changes when the pH decreased from 8.2 to 7.8 [17].

In order to better understand the factors driving hydrolysis patterns, this study investigated the relationship of enzyme activity rates with environmental variables, as well as their spatial and temporal variability. The link of heterotrophic enzyme activity with climatic changes led us to make predictions towards a major consumption and successive utilization of labile organic carbon derived from autochthonous (primary producers) or allochthons sources, so indicating that a heterotrophic metabolism could prevail in future.

## 3. Enzymatic activities

The determination of enzymatic activities is fundamental to quantify microbial contribution to the turnover of organic matter [2]. The process of microbial decomposition is generally considered as a bottleneck in organic matter utilization, because the activities of microbial enzymes determine which substrates are hydrolysed and transported into the cells for further processes or respired and which substrates remain untouched and are transported to other areas or buried into sediments [6],[8].

For the field measurement of enzymatic activities, the fluorimetric method is a widespread technique generally used in aquatic environments, even if specific knowledge about the macromolecular structure of the organic matter in the ocean is lacking. According to the Hoppe's method [30], specific artificial substrates are widely used to measure potential enzyme activities in seawater and sediments. These substrate analogues consist of a monomer [an amino acid or a monosaccharide analogue] linked to a fluorophore, such as L-leucine-7-amido-4-methylcoumarin hydrochloride (leucine-MCA), used to measure leucine aminopeptidase; 4-methylumbelliferyl-β-D-glucopyranoside (MUF-β-glucose), and methylumbelliferyl-phosphate, used to measure β-glucosidase and phosphatase activities respectively. Many other fluorogenic substrates can be used to study chitinase, lipase, α-glucosidase, endopeptidase, ecc. [6].

When the link between the monomer and the fluorophore is cleaved by the specific enzyme, a fluorescent signal is emitted in coincidence with the release of the fluorophore, so that the hydrolysis of the fluorogenic substrate is measured by an increase in fluorescence over time.

Using the multiple substrate concentration procedure, measurements of the velocity of hydrolysis recorded at increasing substrate concentrations allow to calculate the maximum velocity of the reaction (Vmax), that is a measure of the potential enzyme activity of the microbial community present in the sample.

According to the kinetic approach, it is possible to determine also the Michaelis-Menten constant (Km), which expresses the affinity of an enzyme for its substrate [6],[30].

The fluorogenic assay is sensitive and relatively rapid, and the substrates analogues are usually used for comparisons among sites and studies. However, their concentrations routinely used in laboratory hardly reproduce the *in situ* conditions, because *in situ* the substrates are more diversified and their concentrations are generally unknown. A further limitation is that the enzyme assay does not discriminate among the different types of each extracellular enzyme potentially present in the environmental sample and measures only the activities of exohydrolase enzymes which cleave the terminal end of oligomers and not the endo-acting enzymes [2]. Based on these considerations, some recent studies [31] have suggested a conversion of ‘*potential*’ enzymatic activity into ‘*actual*’ rates of *in situ* hydrolysis of the assayed organic polymer.

Microorganisms are able to respond to changes in nutrient and substrate availability in the ecosystems by modifying their enzymatic profiles directly or through changes of microbial community composition; due to the plasticity of microbial metabolism, the determination of enzyme activity rates is considered a good indicator of environmental trophic conditions [9],[17].

During a study of the bacterial enzymatic potential in the north Adriatic Sea in different seasonal conditions, changes of bacterial community were observed. The main enzymatic activities of the bacterial community were peptidases, lipases and phosphatases, with a percentage increase in the hydrolytic potential during summer. Polysaccharides were scarcely hydrolysed by the bacterial community (average 20%) with a different seasonal cycle (alfa-glucosidase was more represented in the cold months, rather than in the warm months). These observations were attributable to succession of bacterial genera that possessed different enzymatic spectra. In fact, many bacterial groups possess peptidase, lipase and phosphatase, while chitinolytic enzymes and glucosidases are present in a limited number of genera only [32]. Other studies with molecular techniques evidenced changes in bacterial assemblage structure between summer and winter-spring periods [33]. However, the organic substances do not have all the same rate of degradation: some, depending on their biochemical composition and molecular structure, are demolished faster, while others require a long time. The lipids are generally the most easily assimilable organic component. The proteins and their derivatives are easily degradable. Carbohydrates make up a large part of the food reserve [8].

Extracellular enzymes can be found associated to cells or dissolved in the surrounding water. The free enzymes, defined as enzymes whose activity can be detected after filtration through a 0.2 µm pore-sized membrane, are recently studied in several environments. In many marine environments cell-free enzymes constitute more than half of the total activity [34]–[37]. They derive from active cells, as a consequence of viral lysis or protist grazing [38]. The importance of free enzymes for carbon cycling depends in part on the temporal and spatial scales over which such enzymes retain activity. These time- and distance scales suggest that these enzymes can affect carbon cycling at sites quite far from their production site [8],[34]. The temperature increase affects also the fraction of free enzymes, reducing the ratio of dissolved to total enzyme activity [36]. Only a few number of investigations, however, have been carried out in the Mediterranean Sea to estimate the contribution of dissolved enzymes to total enzyme activities [36],[37],[40],[41].

The most widespread studies on microbial enzymes in seawater regard the measure of the activities of leucine aminopeptidase -LAP; beta-glucosidase -GLU and alkaline phosphatase -AP.

### 3.1. Leucine aminopeptidase (LAP)

Protein containing particles are very abundant in sea water and constitute a C and N source for bacteria [2],[8],[41].

LAP is an exopeptidase, because it cuts the extreme aminoacid of a polypeptides chain. The total LAP activity is usually used as an indicator of protein hydrolysis, because LAP provides amino acids for cellular metabolism and increases the availability of N; however, the interpretation of LAP activity is complex because this enzyme plays a dual role, being important in both carbon and nitrogen cycles. LAP is an enzyme very widespread in many marine and brackish environments [42]–[45] and may be synthesized by bacteria and cyanobacteria, ciliates and zooplankton [6].

### 3.2. Beta- Glucosidase (GLU)

GLU activity is generally used as indicator of polysaccharide hydrolysis. It is specific for the hydrolysis of cellobiose, a component of polymers such as cellulose and mucopolysaccharides; it contributes significantly to the recycling of both particulate and dissolved organic matter. Differently to LAP, it contributes to Carbon cycle, being able to hydrolyse more refractory organic compounds like polysaccharides and vegetal debris, derived from autochthonous or allochthons production.

Glucosidases are known to be ubiquitously present in seawater [6],[37]. In temperate zones GLU is usually lower than LAP; this can be explained by the presence of constitutive aminopeptidase during summer, when polysaccharides and protein degradation are not correlated [40],[46].

Beside beta-glucosidase, which hydrolyses the beta -D- link of cellobiose, alfa -glucosidase (cleaving alfa linked D-glucose) and more different enzymes are active in polysaccharides hydrolysis, as reported in many studies [7],[45].

### 3.3. Alkaline phosphatase (AP)

Phosphatases are also enzymes widely spread in aquatic environments. AP is involved in the hydrolysis of dissolved organic phosphates (DOP), with regeneration of inorganic phosphate [47]. In the epipelagic layer, the amount of total AP of bacterial origin could also be over-estimated since this enzyme is produced by both prokaryotes (bacteria and cyanobacteria) and eukaryotes (phyto- and zoo- plankton) [39]. AP is usually considered an inducible enzyme, being synthesized under low levels of PO_4_ and repressed when P becomes available. In fact, the expression of ectoenzymes is regulated by a negative feedback, so the availability of inorganic P could have an inhibitory effect on AP synthesis [5]. Therefore, AP has been suggested as an index of P deficiency, because it is produced in great amounts when inorganic P becomes limited [48],[49]. It assumes great importance in very P- limited areas such as the Eastern Mediterranean Sea [49].

To this regard, in the very oligotrophic Levantine Sea a limiting factor for bacterial production was the availability of inorganic nutrients, especially phosphorus. How P limitation could affect microbial food web and influence carbon flow was investigated by Thingstad et al. [50]. However, while phosphorus is usually the limiting nutrient, nitrogen and carbon limitation or co-limitation also occur, and the type of limitation can vary with depth [49],[51]–[53]. Moreover, different mechanisms are involved in the N and P regeneration: AP activity is responsible for the direct mineralization of organic phosphate esters into orthophosphate, while LAP acts in the first steps of protein decomposition, with the release of dipeptides which follow further decomposition pathways before their final mineralization.

The presence of AP below the surface layer and at deep layers assumes a different meaning due to abundance in deep layers of mineralized nutrients such as P. It has been hypothesized that AP is not only regulated by inorganic P concentration, but it could be produced in C-limited conditions, contributing to both C and P cycles [47],[51].

## 4. Ecological features of enzymatic activities

### 4.1. Patterns of distribution in water column, depth gradient

The majority of the investigations of enzymatic activities in marine systems have been carried out in the water column. In marine environments, activities are typically higher in surface waters than at depth and vary between nearshore and offshore sites [26],[27].

Christian and Karl [54] suggested that patterns of peptide hydrolysis may differ between temperate and high latitude sites, increasing at high latitude, while carbohydrate hydrolysis at lower latitudes, similarly to that documented for polysaccharides [45].

The distribution of enzymatic activities in diverse Mediterranean sites, classified into coastal, offshore and frontal systems, is reported in Table 1. Differences in enzyme patterns were observed in relation to time (seasons and years), or space, between coastal and pelagic waters [46],[49]. Consequently, patterns of polymer hydrolysis rates change horizontally and vertically, among different water masses, along estuarine gradients [54]–[57].

Only few studies have considered the distribution of hydrolytic enzymes along longitudinal transects in the Mediterranean Sea: Van Wambeke et al. [51] reported LAP ranging from 1.1 to 9.3 nmol l^−1^h^−1^ without a clear East-West gradient; La Ferla et al. [56] and Zaccone et al [49] along a W-E longitudinal transect (Transmed cruise), found regional differences within a variety of Mediterranean provinces. Also Luna et al 2012 [59] showed significant differences between the West and Central East Mediterranean basins; the variance in LAP activity rates accounted for most of the observed total variance in enzyme distribution.

Aminopeptidase generally showed a decreasing gradient from more eutrophic coastal areas to oligotrophic waters [60]. In the East Mediterranean the range of variation of LAP activity (0.3–4.91 nmol l^−1^h^−1^ [49]) was similar to that reported in the NW Mediterranean [50] and in the Ionian Sea [46],[55], but lower than that observed in the Tyrrhenian Sea [62],[63]. Placenti et al. [64] in an Ionian pelagic area observed high LAP values (Table 1), similar to those recorded in coastal zones [65],[66]. Also in the Atlantic Ocean LAP values varied in a wide range [27],[34].

Subsurface peaks of LAP roughly corresponded to chlorophyll peaks and high rates of activities were observed in the Deep Chlorophyll Maximum (DCM), indicating the contribution of planktonic autotrophic organisms (cyanobacteria and phytoplankton) to the synthesis of this enzyme. The pico- and nano-sized organisms were the dominant fraction in the DCM, with a peculiar microbial food web. The water column stratification during summer resulted in an accumulation of particulate matter, with consequent concentration of prokaryotic biomass and metabolic activity [14],[40].

Generally low hydrolysis rates of GLU were observed in the Central Mediterranean Sea (Table 1), except in autumn along all the water column [63]. Differences in polysaccharide hydrolysis between surface and subsurface waters were observed by Arnostì [45] in other areas, suggesting substantial changes in the spectrum of substrates utilized by microorganisms.

Differences in AP activity levels among the water masses present in the water column were also observed [55]. In the Ionian Sea the decreasing trend of AP *versus* PO_4_ from the surface to deep waters was described by an exponential curve showing a significant correlation (R^2^ = 0.77, p < 0.05) and indicating that the abundance of inorganic P could have an inhibitory effect on AP activity. In the same area an inverse relationship with inorganic P was found [46] except in autumn [64]. Similarly, in the south Adriatic Sea an exponential curve described the vertical decreasing trend of AP *versus* PO_4_ [57]. In the north Adriatic Sea surface AP was significantly higher than at the bottom [37],[38].

In the Ionian Sea the maximum hydrolysis rates of LAP, GLU and AP were measured in the surface layer. Significant differences (ANOVA P > 0.05) among epipelagic layer, meso-, and bathypelagic waters were reported in this basin [46]. Depth profiles of aminopeptidase exhibited surface values 8 times higher than at depth. Several Authors observed this decreasing trend along the water column in relation to availability of organic carbon [49],[55],[56],[64],[67]. Also Luna et al. 2012 [59] showed significant differences among epi- meso- and bathypelagic layers (P < 0.001). Occasionally, an increasing trend of LAP towards deep layers was observed [63].

Heterotrophic microbes at depth are able of degrading organic matter (semi-labile DOC) that resists to the biological removal occurring in the surface ocean [45]. Additional deep sea measurements are from Misic and Fabiano [68] and Celussi et al [69]. A strong relationship linking enzyme activity rates with the presence of different water masses is a common finding of these studies [46],[49],[55],[57].

Depth profiles of bacterial abundance and metabolic rates extending to the deeper meso- and bathypelagic zones indicate that cell numbers decreased by a larger factor than enzyme activities, so that cell-specific enzyme activities were calculated to really compare with measurements taken in surface the observations obtained from deeper waters. These studies also found that enzyme activities calculated on a per-cell basis were generally higher at depth compared to surface ocean waters; this approach did not take into account the amount of active or non-viable cells, nevertheless it allowed to compare different environments [34].

The vertical distribution of enzyme activities normalized per cell showed a trend different for the three enzymes: LAP prevailed in the epi- and bathypelagic layers, whereas the importance of GLU increased at the mesopelagic layer [46]. In the deep zone of the Ionian Sea also Placenti et al [64] observed an increasing trend in the specific activity of deep prokaryotes; the importance of specific GLU activity, compared with LAP, indicated a preferential hydrolysis of more refractory material, such as mucopolysaccharides, compared to the protein fraction, which was preferentially hydrolysed in the surface layers [46],[63]. An increase in the specific enzyme activity with depth was also observed by Baltar et al. [67] who explained this result as an adaptation of enzyme activity to exploit the scarce amount of available organic matter or to its refractory nature.

### 4.2. Patterns of temperature, seasons, fronts

The sea temperature increase in the Mediterranean Sea may have effects on heterotrophic microorganisms, and could reinforce the major role in organic matter decomposition or production, alternatively, in the carbon cycle [25]. According to Wohlers et al. [22], the temperature increase detected in surface waters affected the hydrological structure of water column, favouring water stratification and stimulating the biological processes. Weather dependent alterations of seawater temperature with implications on bacterial metabolism were also observed [26],[70]. As most of the extracellular enzymes are adaptive, environmental and biological factors such as temperature, pH and salinity, quality and quantity of organic matter may affect their activity rates. Physical and chemical factors partially explain the changes in potential enzymatic activities. This does not mean that necessarily there is a direct causal effect, but multiple factors concur to stimulate bacterial activity. The correlative approach led us to hypothesize that the increase of microbial processes on organic matter was linked to water warming. The meaning of this finding could be that the rising temperatures, as expected by water warming, may cause an increase in remineralisation–at the cost of photosynthetic production – with consequent predominance of a heterotrophic regime [26]. In fact, increasing temperature stimulates microbial growth, but cells require also substrate and nutrients, supplied by microbial activities.

**Figure 1.**
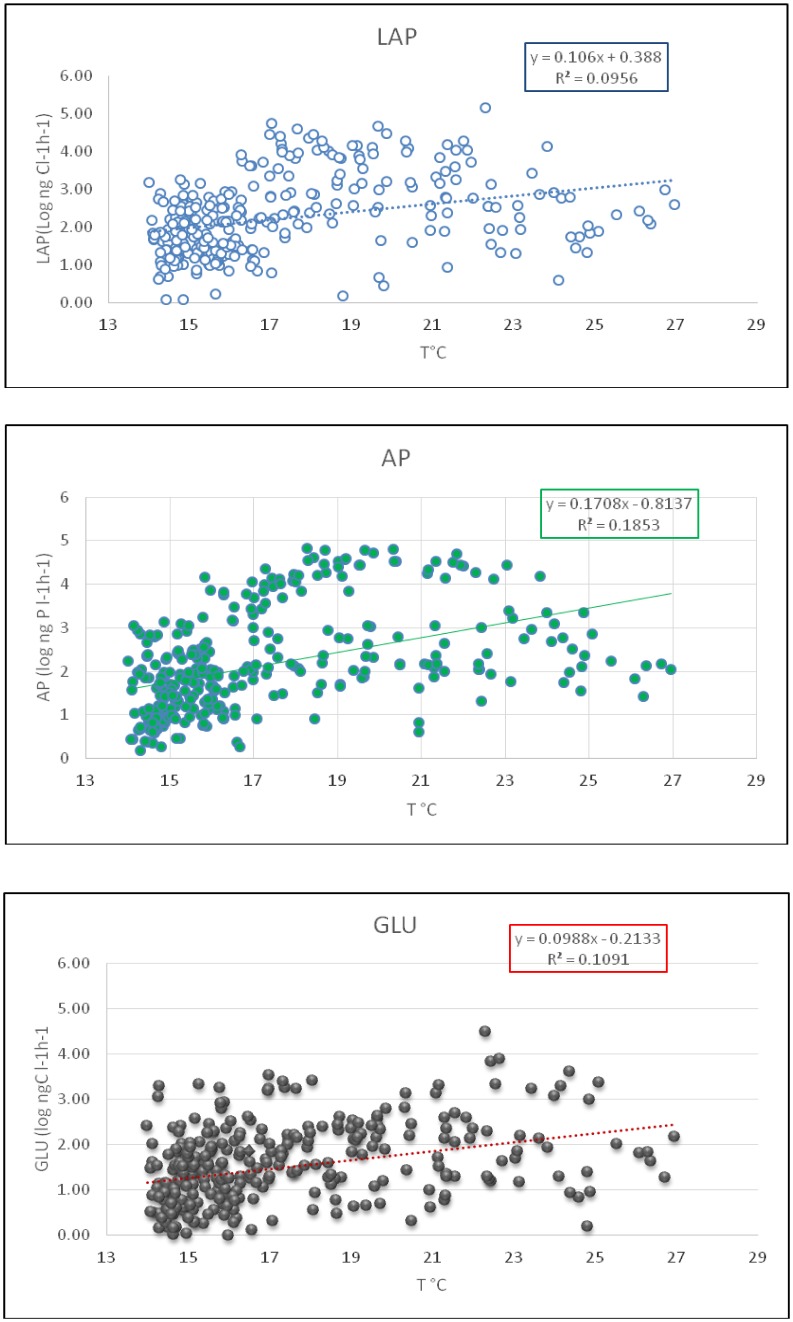
Temperature in the euphotic layer of the Central Mediterranean *versus* LAP, AP and GLU activity rates from 1999 to 2013 (n = 320, P < 0.001).

The surface layer was particularly sensitive to temperature changes and seasons. In Figure 1 the enzymatic activities were plotted in relation to water temperatures in the epipelagic layer of Ionian Sea. The relationship was significant for all tested enzymes, with different slopes. In the meso- and bathy-pelagic layer the relationship with temperature was not significant, because the range of T varied from 13.30 to 15.00 °C, over the different seasons.

Moreover, the warming effects on microbial components have been explored in the Sicily Channel in relation to fishery resources. The results confirmed that a co-variation of prokaryotic activities and eggs distribution with temperature exists in summer, suggesting a reciprocal relationship among them [14],[40].

Different behaviour in prokaryotic abundance and microbial enzymatic activities between cold and warm periods was previously observed [46],[71]. Conversely, during winter the effect of T was scarce, because the epipelagic layer was mixed (ΔT = 0.13 °C). In this season the recycling of P by microbial activity assumed more importance in controlling prokaryotic biomass and activities [40]; this was in agreement to Cea et al. [71] that observed P limitation in the NW Mediterranean Sea.

In coastal and estuarine environments, the abiotic factors that influence microbiological processes may be the same, but change in their relative importance depending on the type of environment. However, particular elements can be recycled more quickly than others, causing decoupling in biogeochemical cycles. In these ecosystems the effect of T increase was higher than in pelagic areas, indicating a higher degree of correlation between bacterial activity and temperature. There was also a narrow co-variation with bacterial production and chlorophyll-a content [27],[42].

In the superficial waters of the Adriatic Sea a doubling of the peptidase activity has been observed in summer in comparison to the spring [70],[72]. Coastal ecosystems - which are subject to rapid changes of organic matter due to main current, riverine input, or eutrophication - support a wide spectrum and high rates of enzymatic activity [32],[43],[74]. In fact, in these environments micro-organisms respond rapidly to changes in nutrient and substrate availability modulating their enzymatic profiles in relation to new organic polymers and, in turn, changes in the specific composition of the microbial community affect the decomposition process [45].

The distribution of enzymatic activity and in particular of proteases in different marine and lacustrine environments highlighted a great activity in the superficial layers. An increase in enzyme activity may also occur near the bottom in relation to the available organic matter. In brackish waters, changes in prokaryotic metabolism did not depend on variations in the prokaryotic abundance, but rather on the stimulating effect played by seasonal trophic variations. In these small ecosystems seasonal changes in the local environmental parameters (trophic supply, CHL, POC, allochthons input, storm events) are more evident and significant seasonal variations in the patterns of microbial activity in relation to temperature, dissolved oxygen and trophic changes were found [45],[74]. The measured rates of enzymatic activities were consistently higher than that observed in coastal marine areas; moreover, positive correlations between temperature and LAP, AP, particulate organic matter were observed in Ganzirri Lake [45],[75].

### 4.3. Salinity gradient and brackish coastal zones

Salinity is a significant factor affecting negatively the enzyme distribution in most areas of the Mediterranean Sea [49]. The effect of Salinity is due to other changes (like inorganic nutrient input, land derived organic load, primary production stimulation etc.) that the river water causes in coastal waters. [66],[70],[72].

A negative correlation among S and abundance and microbial activities was reported in several aquatic environments [17],[31],[43],[58],[72]. In fact, in the Adriatic Sea, where inputs of the Po river affect a wide coastal zone, the range of salinity was very different from that of pelagic areas. The LAP activity was concentrated in the surface layer where the stratification of the fluvial waters brings high amounts of organic matter, and decreased along a gradient of salinity from the coastal stations towards the open sea [73]. The activity values were 1.5–1.7 times greater in summer than in winter [71], with a trend that followed the trophic gradient towards the Po delta.

On the contrary, in the pelagic area of Sicily Channel salinity did not appear to be one of the most important physical-chemical parameters driving microbial metabolism and abundance. The salinity range measured in the Ionian Sea by Zaccone et al. [40] was very small (ΔS = 0.72 in summer and 0.76 in winter), like that observed by Monticelli et al. [58] in a coastal zone affected by a thermohaline front (ΔS = 0.81, ΔT = 3.44 °C).

In brackish environments a gradient of salinity and progressive segregation from the sea was observed in the coastal lagoon of Oliveri-Tindari, a very suitable study environment with ecological characteristics differing from each other (Marinello ponds) [60]. The marked spatial and temporal diversification of the ponds in relation to their trophic features (ranging from oligo- to eutrophic) was reflected in a various and unpredictable range of enzyme patterns. With respect to the ponds nearest to the sea, higher level of microbial activity (ranges: 0.55 to 4359.03 nM h^−1^, 0.15 to 76.44 nM h^−1^, 1.29 to 1548.06 nM h^−1^ for LAP, GLU and AP respectively) and a greater variability, due to seasonal course and terrestrial input, characterised the most ‘confined’ ponds. Lower enzyme activities were observed in less eutrophicated ponds. With regard to their seasonal distribution, all the enzymes examined showed a sharp increase in all ponds from March to June (spring peak), and a second peak in August–September [60].

In the Cape Peloro lagoon AP was 10.83 folds higher in summer-autumn than in winter-spring, while GLU in summer-autumn was 19.6 folds higher than in winter-spring. Moreover, during summer-autumn periods a prevalence of AP over the LAP and GLU activities was observed, indicating the high demand of P, that was quickly regenerated. On the contrary during winter-spring months the prevalence of LAP over other enzymes confirmed changes in prokaryotic metabolism [75].

### 4.4. Enzymatic stoichiometry and biogeochemical implications

The relative importance of LAP, GLU and AP reflects changes in the relative amounts of proteins, polysaccharides and organic phosphates, respectively. In surface waters, the magnitude of enzymatic activities is generally in the order aminopeptidase > phosphatase > glucosidase. A more rapid hydrolysis of leucine-MCA compared to the one of MUF-glucose indicates a more rapid remineralization of organic nitrogen than organic carbon during early diagenesis [2],[8]. This order of magnitude may change in relation to season, latitude (polar or temperate zones), quality of available organic matter in water column or sediments [17],[54]. Variation in the relative activities of different enzymes is suggested as a possible indicator of changes in the modalities of bacterioplankton nutrition [54].

The reciprocal ratios among enzyme activities in plankton communities have been focused by some Authors [76] who have suggested the ‘ecoenzymatic stoichiometry’ as a tool to connect the elemental stoichiometry of microbial biomass and organic matter to nutrient assimilation and microbial growth. According to this approach, the relative abundance of enzymes involved in C, N, and P cycles reflects the biogeochemical equilibrium between microbial biomass and the elemental composition of organic matter.

The reciprocal ratios between enzymatic activities (LAP/GLU and LAP/AP) reported by different Authors are shown in Table 1.

**Table 1.** Selected examples of enzymatic rates measured in different areas of the Mediterranean Sea. *range or mean values (in bold ratio <1).

Area	Depth (m)	Sites	season	T °C	S	LAP(nM h^−1^)*	GLU(nM h^−1^)*	AP(nM h^−1^)*	LAP/GLU molar ratio	LAP/AP molar ratio	References
NW Mediterranean	10–2000	DYFAMED area	spring	>12.70	38.40	1.78–0.23		1.12–0.22		1.6	[61]
	10–2000	DYFAMED area	fall			1.44–0.45		1.5–1.2		**0.37**	"
NW Mediterranean		DYFAMED area	autumn 04			3.7–4.6		2.4–3.2			[53]
Mediterranean	5–200	W-E transect	summer 99	22.7–24.9	37.28–39.01	1.1–9.3		0.62–1.65			[51]
NW Mediterranean	5	Marseille Bay	annual 12-13	12.8–25.8	38.15	5.1–85.1		0.1–115.7		5.42	[71]
Ligurian Sea	5–200	gulf of Genoa	summer 02	17–24	>36.7	1.78–4.04	0.04–0.19	1.52	25-68		[68]
Ligurian Sea	0.5	Portofino	spring -summer 07	19.4		110.1–165.6		13.9–29.4			[66]
South Tyrrhenian Sea	1–100	Eolian Island	spring 04			1.9–2.5	0.04–0.08				[65]
Tyrrhenian sea	25–3000		winter 05	13–16	37.8–38.25	0.3–21.7		0.5–9.9			[62]
South Tyrrhenian Sea	5–100		summer 05			17.55	0.59	3.60	29.75	4.88	[63]
"	200–1000		"			23.71	0.80	0.78	29.64	30.40	"
"	>1000		"			37.72	0.40	0.93	94.3	40.56	"
Tyrrhenian sea	10–200	st. VECTOR	summer 07	16.79	38.08	0.31	0.31	1.16	1	**0.27**	[49]
"	500–1000	"	"	13.81	38.69	0.37	0.37	0.19	1	1.95	"
"	1500–3500	"	"	13.49	38.53	0.61	0.47	0.20	1.29	3.05	"
Ionian Sea	5–200	pelagic	winter 98	15.17	38.59	1.92	0.19	1.30	9.91	**0.67**	[55]
	400–1000		"	13.98	38.80	1.59	0.16	0.16	9.94	**0.10**	"
	1100-4000	pelagic	"	13.92	38.76	0.51	0.06	0.92	8.81	1.79	"
Ionian Sea	5–200	pelagic	winter 99	15.79	38.65	5.40	4.85	9.97	1.12	**0.54**	[26], [46]
	400–1000		"	13.60	38.73	2.83	0,70	1.90	4.05	1.49	"
	2000–3300		"	13.39	38.71	5.97	0.49	11.97	12.17	**0.50**	"
Ionian Sea	5–200	pelagic	autumn 00	17.47	38.68	1,48	1,79	99.86	**0.83**	**0.02**	[26],[46]
	400–1000		"	13.77	38,76	1,58	0,51	52.27	3.11	**0.30**	"
	2000–3300		"	13.79	38.73	1,53	2,86	35.41	**0.53**	**0.04**	"
Ionian Sea	5–200	pelagic	summer 01	17.38	38.62	2,02	0,20	5,08	10.12	**0.40**	[26],[46]
	400–1000		"	13.81	38.76	10,64	0,35	4,82	29.97	2.21	"
	2000–3300		"	13.79	38.73	1,08	0,34	3,12	3.17	**0.35**	"
Ionian Sea	5–200	pelagic area	autumn 04	17.14	38.44	4,95	2,04	4,06	2,43	1,22	[26],[46]
	500–1000		"	13.96	38.80	3,60	0,34	1,63	10.46	2,21	"
	1100– 4000		"	13.85	38.80	4,53	0,80	2.01	5.67	2,25	"
Ionian Sea	5–200	pelagic area	spring 02	15.49	38.58	2,96	1,44	5.29	2.05	**0.56**	[26],[46]
	500–1000		"	14.02	38.79	0,50	0,10		4.87	**0,97**	"
	1100– 4000		"	13.84	38.74	0,33	0,09	0,16	3.70	2,03	"
Ionian Sea	0–150	pelagic area	autumn 11	>15.50	<38.80	21.10	18.91	144.99	1.12	**0.15**	[64]
	200-600	"	"	< 15.00	>39.00	20.84	15.03	14.26	1.39	1.46	"
	>3000	"	"	13.42	>38.72	20.39	12.94	12.21	1.58	1.67	"
Ionian Sea	5–100	pelagic area	summer 12	18.16	38.29	4.91	1.37	2.04	3,59	2,40	[26],[40]
Ionian Sea	5–100	"	winter 13	14.97	38.45	1.49	0.27	16.74	5.53	**0.09**	"
Ionian Sea	1–60	coastal station	autumn 01	19.26	38.53	207.29	11.28	717.8	18.37	**0,29**	[79]
Atlantic ocean	5–100	Atlantic station	summer 07			0.38	0.27	0.22	1.41	1.73	[49]
	200–1000		"			0.74	0.13	0.05	5.69	14.8	"
	1500–3000		"			0.57	0.25	0.01	2.28	57	"
West Mediterranean	5–200	pelagic area	summer 07	16.21	37.8	0.35	0.20	1.21	1.75	**0.29**	[49]
	500–1000		"	13.36	38.57	0.21	0.32	0.19	0.66	1,11	"
West Mediterranean	1500–3500		"	13.29	38.50	0.36	0.25	0.20	1.44	1.8	"
East Mediterranean	5–200		summer 07	18.41	38.79	1.53	0.50	4.56	3.06	**0.33**	[49]
	500–1000		"	14.01	38.81	0.66	0.52	0.33	1.27	2.01	"
	1500– 2900		"	13.95	38.75	1.22	0.58	0.78	2.10	1.56	"
West Mediterranean	5–200					2.18	0.88	0.51			[59]
	500–1000					1.04	0.51	0.35			"
West Mediterranean	1500–3500					0.66	0.41	0.31			"
East Mediterranean	5–200					0.08	0.31	0.20			[59]
	500–1000					0.09	0.10	0.13			"
	1500– 2900					0.05	0.13	0.03			"
North Adriatic Sea	gen-60	coastal station	seasonal study			2.2–1701	0.3–114	0.8 -1770			[70]
North Adriatic Sea	1–60	coastal station	spring-summer			115– 67.5	2.1–5.2	13.6	22.14	4.96	[73]
North Adriatic Sea			autumn					2.04–4.64			[38]
North Adriatic Sea	1–10	coastal station	spring-autumn					70–4640			[37]
South Adriatic Sea	5–100	convection site	seasonal study	13.8–15.6	38.71–38.74	0.01–7.86	0.003–3.5	0.05–31.96			[57]
	100–800		"	13.6–13.6	38.74–38.77	0.01–2.66	0.003–0.98	0.003–6.47			"
"	800–1200		"	12.9–13.1	38.72–38.73	0.01–0.15	0.003–0.95	0.08–17.96			"
South Adriatic Sea	0–30	Gulf of Manfredonia	spring 03	18.44	37.92	13.99	1.13	43.32	12.4	**0.32**	[58]
	0–30	offshore waters	"	17.25	38.73	31.18	0.48	43,28	72.51	**0.72**	"
	>30	"	"	14.11	38.70	19.45	0.26	23.49	75.97	**0.83**	"
North Atlantic	surface					4.2–24.9					[27]
South Atlantic	surface					11.5–151.4					"
Atlantic ocean	100–4500					0.2–13.8	0.05–0.25	0.14–1.3			[34]
subtropical Atlantic	100–4500					0.6–9.2	0.02–0.07	0.04–3.7			[67]

Particularly, the LAP/GLU ratio indicates the ratios between the two processes of proteolysis and glycolysis and depends on the functional features of each ecosystem. High LAP/GLU ratios provide information on the flux of organic matter preferentially through proteins, while low ratios indicate a preferential flux of organic matter through polysaccharides [44]. Together with the common quality indexes (C/N ratio), this ratio provides information on the potential lability of the organic matter [44],[66].

LAP/GLU ratios higher than 1 are generally observed in coastal and pelagic environments and suggest the presence of fresh organic matter such as those produced by phytoplankton [2],[48]. In the Mediterranean Sea, positive ratios have been used as an indicator for nitrogen-rich *versus* carbon-rich substrates [68] (Table 1).

In the Ionian Sea, the LAP/GLU as molar ratio ranged from 0.8 to 10.1 in epipelagic waters, with high values in summer, indicating that microbial community uses LAP to have access to protein substrates as both C and N source [46],[64]. Along a Western-Eastern Mediterranean transect Zaccone et al [49] found the prevalence of LAP over GLU along a West–East transect in epi- and bathy-pelagic layers (Table 1).

High LAP/GLU ratios in deep Ionian waters were found in winter 1998 and 1999 [46],[55]. A seasonal trend of this ratio was observed by Azzaro et al [57] in the south Adriatic Sea, with higher values in April and June probably due to a high amount of proteins in the photic layer during productive phase. LAP/GLU ratio decreased during phytoplankton decay and the protein components of organic matter were more rapidly degraded during particle sinking [57].

This ratio was calculated also in the north Tyrrhenian Sea [65]. In the Central Tyrrhenian Sea higher LAP/GLU ratios in the bathypelagic layer than in the other ones were reported, suggesting high metabolic potentialities of prokaryotes in the degradation of proteinaceous material in the deep realm [63]. Also Monticelli et al. [58] observed high values of this ratio in a frontal system in the southern Adriatic Sea (Table 1).

In marine aggregates different enzymatic ratios and an imbalance in bacterial enzymatic activity, with high values of protease activity and relatively low values of glucosidase activity, resulted in a progressive increase in the refractory component of the organic matter and a consequent increase in its C/N ratio [78]. The incomplete hydrolysis of organic matter related to low enzyme specificity lead to increasing recalcitrance and accumulation of long-lived organic matter, which could aggregate into mucilage [70].

In the brackish waters of Ganzirri lake the decomposition of polysaccharides was enhanced during post-bloom and the LAP/GLU ratio increased from 1.18 in summer–autumn period to 7.17 during winter–spring [75].

The molar ratio between AP and LAP was proposed by Sala et al [48] to assess nutrient limitation in microbial population, being its values high when P and N are unbalanced.

AP/LAP ratios measured in the Ionian Sea during our previous studies showed different distribution patterns: a prevalence of AP over LAP and GLU was observed in the epipelagic layer and LAP/AP ratio was lower than 1, thus indicating that that microbial community was P-limited in surface layer (Table 1). In autumn 2000 similar low values were measured in all layers [46], suggesting that AP supplied the P necessary for protein synthesis, cell production and energy transport for both bacteria and phytoplankton [40],[71].

In the West and East Mediterranean waters the LAP/AP ratios were lower than 1 at surface during summer, indicating the quick remineralization of organic phosphates with prompt recycling of P in this layer [49]. In the surface layer of oligotrophic areas, a low ratio between LAP and AP, as proxy of P-limitation, suggested that bacteria are P limited, while the relative importance of AP decreased with increasing depth (Table 1). Also during spring and winter the needs of P for the primary production increased, so the LAP/AP ratio decreased [46],[58]. On the contrary in the Tyrrhenian waters the LAP/AP ratios showed values >1 both at surface and at high depths [63]. Deep waters were rarely P limited (Table 1), with some exceptions [55] where P limitation was found in both the deep and benthic boundary layers (LAP/AP = 0.4 and 0.6, respectively).

In the north Adriatic Sea, seasonal differences in the AP activity with high values in summer period were observed by Ivancic et al. [37] in both waters and aggregates. In a seasonal study, AP prevailed over LAP during winter (LAP/AP = 0.09) suggesting that P was more efficiently recycled in this season than in summer; in winter, the high AP activity contributed to P needs supporting prokaryotic growth [40].

Also in the DCM a rapid recycling of P with respect to N and C was found in the Augusta bay [79].

## 5. Perspectives

The Mediterranean Sea offers then an attractive marine environment to study general ubiquitous processes across multiscale and multidirectional physical, biological and trophic gradients. Despite the numerous investigations of the last decades, the emerging picture of enzyme dynamics in this ecosystem is far from being satisfying, both at spatial and temporal scales. Some areas, especially in both the West and East Mediterranean basins, are still insufficiently known. More information is available in the Central Mediterranean Sea, where the relationship with temperature changes is evident [26]. At the epipelagic layers and in coastal zones an increase in the decomposition processes has been detected.

The temporal variability at seasonal and interannual scale also needs more intensive sampling and long-term records to investigate seasonal patterns and responses of the planktonic microbial metabolism to anthropogenic and climatic changes.

Although the relationships between climate and microbial processes need to be elucidated yet [27], in a warmer ocean microbial processes are expected to play an even more important role in the carbon cycle. In the context of climate changes, the fate of bacterial metabolism will depend on the rates at which labile organic carbon derived from primary producers will become available to incorporation into prokaryotic cells. However, the prediction of metabolic rates and the partitioning of their carbon uptake towards autotrophy or respiration by heterotrophs remain uncertain [18]. Long term studies on microbial activity and abundances with rising temperatures are needed to describe the evolutionary scenario and future biogeochemical implications of microbial processes in the Mediterranean Sea.
